# Hypocalcemic cardiomyopathy: A case report

**DOI:** 10.3389/fcvm.2022.999550

**Published:** 2022-09-13

**Authors:** Yi Wen, Xiaolin Luo

**Affiliations:** ^1^West China School of Nursing/West China Hospital, Sichuan University, Chengdu, China; ^2^Department of Cardiovascular Medicine, West China Hospital, Sichuan University, Chengdu, China; ^3^Department of Cardiology, Xinqiao Hospital, Army Medical University, Chongqing, China

**Keywords:** hypocalcemic cardiomyopathy, hypocalcemia, hypoparathyroidism, heart failure, thyroidectomy

## Abstract

Hypocalcemia and its related symptoms are common manifestations in postsurgical hypoparathyroidism, but patients with hypocalcemia manifested as heart failure is rare and few cases are reported in the literature. Here we reported a 58-year-old female with hypoparathyroidism and uncontrolled hypocalcemia after thyroidectomy, presented with acute heart failure, accompanied with enlargement and reduced ejection fraction of left ventricle. She was refractory to guideline-directed medical therapy for heart failure including digitalis and diuretics. However, her symptoms resolved and cardiac function improved dramatically after normalization of serum calcium level. This rare case highlights the pivotal role of calcium in maintaining cardiac function and the importance of treating underlying reversible causes of heart failure. For patients with hypoparathyroidism, it is essential to get standard treatment to avoid development of heart failure and hypocalcemia related syndromes.

## Introduction

Thyroidectomy is one of the main reasons of hypoparathyroidism (HypoPT) (1). Manifestations of HypoPT include hypocalcemia, hyperphosphatemia, neuromuscular excitability, and ectopic tissue calcification (1, 2). However, hypocalcemia manifested as heart failure after thyroidectomy is quite rare (3). Besides, previous cases indicated that traditional therapy for heart failure, including diuretics and digitalis, is not effective in resolving these patients' symptoms (4). Here we reported a patient presented with left ventricular dysfunction induced by hypocalcemia after thyroidectomy, which recovered after normalization of serum calcium. Therefore, clinicians should seek and treat any underlying reversible causes of heart failure to improve the prognosis (5).

## Case presentation

This is a 58-year-old female who was admitted to our hospital with the complaint of exertional dyspnea for 3 months. She underwent total thyroidectomy 2 years ago for papillary thyroid carcinoma in another tertiary hospital. Two days after surgery, laboratory tests showed a decreased serum concentration of parathyroid hormone (PTH) of 2.64 pg/ml [reference range (Ref): 15–65 pg/ml], concomitant with a total calcium of 1.86 mmol/L (Ref: 2.2–2.65 mmol/L) and a phosphate of 1.54 mmol/L (Ref: 0.81–1.55 mmol/L). Then she was treated with Calcium Gluconate Oral Solution, Calcium Carbonate and Vitamin D3 Tablets and alfacalcidol. Meanwhile, levothyroxine (100 ug per day) was given for supplementation of thyroxine. After discharge, however, she did not come to hospital for test of serum calcium, phosphate and PTH level. What's worse, Calcium Carbonate, Vitamin D3 Tablets, and Alfacalcidol were withdrawn on her own. Six months after surgery, she started to suffered from paroxysmal tetany, which could be relieved by calcium supplementation. However, she did not seek for further medical intervention. Three months before admission, she began to experience exertional dyspnea and shortness of breath, accompanied by nocturnal paroxysmal dyspnea and orthopnea. Cardiac ultrasound at local hospital found an enlarged heart and a decreased left ventricular ejection fraction (LVEF). She was diagnosed with heart failure and treated with diuretics and guideline-directed medical therapy (GDMT) for heart failure with reduced EF, but her symptoms did not resolve. She has no history of hypertension, diabetes mellitus, coronary heart disease, valvular heart disease, congenital heart disease, no history of alcohol abuse and tobacco smoking, and no family history of cardiomyopathy. She underwent a laparoscopic cholecystectomy for gallbladder stones 6 months ago.

Physical examination revealed a blood pressure (BP) of 109/92 mmHg, a respiration rate of 24 breaths per minute, and a heart rate of 124 beats per minute. The jugular veins were normal. Pulmonary auscultation found fine rales at the base of both lungs. The cardiac auscultation found a diminished S1 and S2 without any murmurs. Mild pitting edema was found in lower limbs. Chvostek sign and Trousseau sign were negative.

**Figure 1:**
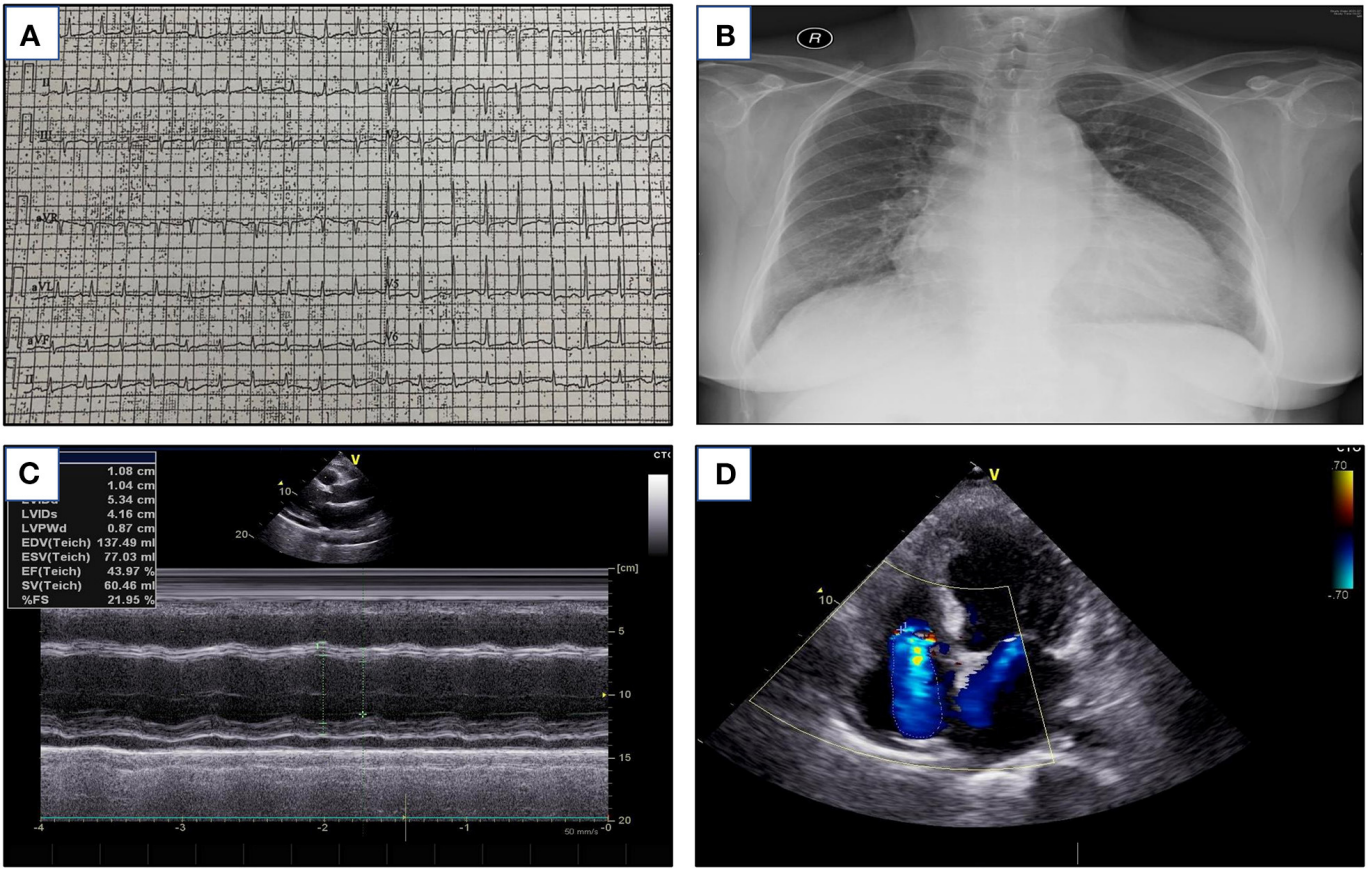
**(A)** The ECG at admission (speed 25 mm/s, 10 mm/mV) with HR 128 beats/min and QTc interval of 502 ms. **(B)** The chest radiography shows cardiac enlargement and signs of pulmonary congestion. **(C)** The echocardiography in the long-axis, M mode shows an increased left ventricular end-diastolic diameter (5.0 cm) and reduced EF (43%). **(D)** Doppler ultrasound shows moderate mitral regurgitation and severe tricuspid regurgitation.

On admission, laboratory tests revealed brain natriuretic peptide (BNP) was 1200 pg/ml and cardiac troponin I was negative. Serum total albumin-corrected and ironized calcium concentration was decreased, and phosphate concentration was increased. PTH was 44.30 pg/ml. Thyroid hormones concentrations were within normal range (summarized in Table 1). Electrocardiogram (ECG) showed sinus tachycardia and prolonged corrected QT interval (QTc) of 502 ms (Figure 1A). The chest X-radiography found enlargement of the heart (Figure 1B). Echocardiography found dilated left ventricular diameter of 50 mm, accompanied by moderate mitral regurgitation, severe tricuspid regurgitation and global hypokinesis of left ventricular wall with LVEF of 43% (Table 1; Figures 1C,D). Coronary computed tomography angiography showed no abnormalities of coronary arteries. Cardiac magnetic resonance imaging was planned but the patient refused. Other causes of hypocalcemia besides HypoPT were not identified.

**Table 1:** Laboratory values and echocardiography parameters.

	**At admission**	**During admission**	**Before discharge**	**2021.9.5**	**2022.1.17**	**2022.3.17**	**2022.8.1**	**Reference range**
Calcium total, mmol/L	1.84	1.93	2.38	2.19	2.08	2.33	2.27	2.02–2.6
Calcium ionized, mmol/L	0.92	-	1.07	1.10	-	1.16	-	1.12–1.23
Albumin adjusted calcium, mmol/L^#^	1.87	-	2.44	2.03	1.93	2.13	2.21	-
Phosphate, mmol/L	1.98	2.16	2.15	-	1.74	-	1.45	0.9–1.34
Magnesium, mmol/L	0.74	0.92	1.10	-	0.99	-	-	0.65–1.05
PTH, pg/ml	44.30	35.80	8.20	-	4.50	-	-	12–65
25-OH vitamin D, ng/ml	22.10	21.80	17.90	-	-	-	-	30–40
Calcitonin	<2.0	<2.0	<2.0	-	-	-	-	0–5
BNP, pg/ml	1,200	-	544	200	46.54^*^	-	46.6^*^	5–100
ALT, IU/L	104.1	-	33.7	23	13.6	18	23	7–40
AST, IU/L	62.31	-	33.2	33	17.6	16	23	13–35
Albumin, g/L	38.5	-	37.1	47.9	47.5	49.8	43.1	40–55
Creatine, umol/L	91.1	-	80.4	77.0	73.0	62.0	62.6	45–105
FT3, pmol/L	2.74	-	-	2.36	3.53	2.153	-	2.43–6.01
FT4, pmol/L	19.04	-	-	20.28	23.12	15.94	-	9.01–19.05
TSH, mIU/L	2.635	-	-	4.16	14.90	13.32	-	0.35–4.94
T3, nmol/L	0.83	-	-	0.99	-	0.979	-	0.98–2.33
T4, nmol/L	138.37	-	-	93.63	-	99.21	-	62.68–150.84
Echocardiography								
EDD, mm	50	52	-	51	40	-	-	-
LVEF, %	43	38	-	41	53	-	-	-
MR	Moderate	Moderate	-	Mild	None	-	-	-
TR	Severe	Mild	-	Mild	None	-	-	-

PTH, parathyroid hormone; BNP, brain natriuretic peptides; ALT, alanine transaminase; AST, aspartate transaminase; FT3, free triiodothyronine; FT4, free thyroxine; TSH, thyroid stimulating hormone; T3, triiodothyronine; T4, thyroxine; EDD, end-diastolic diameter; LVEF, left ventricular ejection fraction; MR, mitral regurgitation; TR, tricuspid regurgitation.

* NT-proBNP.

# Adjusted calcium concentration (mmol/L) = total calcium (mmol/L) +0.02*(40-albumin in g/L).

Based on these findings, acute heart failure resulting from hypocalcemia and chronic HypoPT were diagnosed. Since loop diuretics may exacerbate the loss of calcium from urine (2), hydrochlorothiazide (25 mg per day) and spironolactone (20 mg per day) were prescribed instead to relieve heart failure syndrome. The 10% calcium gluconate injection was administered intravenously (10 ml per day, 3 days; 90 mg elemental calcium/10 ml) and calcium gluconate oral solution (20 ml, three times per day; 90 mg elemental calcium/10 ml) was given, accompanied by alfacalcidol (0.5 ug per day) to correct hypocalcemia. According to the guidelines for heart failure, Sacubitril Valsartan Sodium Tablets and Metoprolol Succinate Sustained-release Tablets were also given and dosages were titrated to the most tolerance (5, 6). From the time of discharge till 1 month after discharge, the drugs contained calcium gluconate oral solution (20 ml, three times per day), alfacalcidol (0.5 ug per day), sacubitril valsartan (titrated to 100 mg, twice a day), metoprolol (titrated to 142.5 mg per day), hydrochlorothiazide (25 mg per day) and spironolactone (20 mg per day). Since the patient still experienced sinus tachycardia during the titration of metoprolol, ivabradine (7.5 mg, twice a day) was initially combined, and the dosage of levothyroxine (initially 100 ug per day) was simultaneously reduced (75 ug per day). Before discharge, dyspnea and other heart failure syndromes were relieved, albumin adjusted calcium increased to 2.44 mmol/L and BNP decreased to 544 pg/ml.

One month after discharge, the patient experienced an episode of sinus bradycardia, ivabradine was withdrawn and heart rate returned to 70–80 beats per min. She underwent regular monitoring of serum calcium and phosphate. Six months after discharge, she was asymptomatic and could tolerate daily activities. Moreover, she had no longer suffered from episode of tetany. Hypocalcemia and hyperphosphatemia were corrected, the albumin-adjusted calcium was 2.21 mmol/L and serum phosphate was 1.45 mmol/L. ECG showed sinus rhythm with normalized QTc interval of 440 ms. Echocardiography revealed normalization of left ventricular diameter to 40 mm, no mitral and tricuspid regurgitation, improvement of LVEF to 53%, and mild hypokinesis of left ventricular wall. Sonography of urinary tract was performed and no kidney stones were found. Now, the patient is still taking the medications at the time of discharge except hydrochlorothiazide and ivabradine and is being closely followed up.

## Discussion

Although prognosis of patients with heart failure has been improved with novel evidence-based therapy, many patients still died of advanced heart failure. Early intervention of reversible factors of heart failure, like ischemia and valvular heart disease, could greatly improve the prognosis. Therefore, it should always be emphasized to assess and treat reversible causes for each individual with heart failure (5, 6). In the present case, the patient suffered from serious heart failure symptoms but was initially refractory to traditional therapy (e.g., digitalis and diuretics). Considering the history of total thyroidectomy, episode of paroxysmal tetany, hypocalcemia, hyperphosphatemia and inappropriate “normal” PTH level on admission, chronic HypoPT was diagnosed (2, 7). Dramatically, symptoms of heart failure and LVEF improved gradually and restored to normal with the correction of hypocalcemia. Therefore, the diagnosis of hypocalcemic cardiomyopathy of our patient was made according to the criteria proposed previously (4, 8).

In most cases, hypocalcemic cardiomyopathy manifests as a treatable cause of heart failure as normalization of calcium concentration could lead to complete recovery of cardiac function (3, 4, 9–12). However, prolonged and profound hypocalcemia may cause irreversible myocardial damage and extensive interstitial fibrosis in rare cases, in which complete recovery of cardiac function could not be reached (13, 14). In addition, discontinuation or poor compliance of calcium supplementation therapy can result in recurrence of the disease (3, 15).

The mechanism of hypocalcemic cardiomyopathy is thought to be related to the vital role of calcium in myocardial excitation-contraction coupling (16). Besides, hypomagnesemia, low PTH and vitamin D level in chronic HypoHT are also assumed to participant in the development of the disease (17–19). Thus, hypocalcemia may lead to left ventricular systolic dysfunction. It is reported that 98% of the cases present with reduced LVEF published in the literatures (3). On the other hand, calcium channel blockers may worsen heart failure or increase late onset congestive heart failure in postinfarction patients due to its negative inotropic effect (20, 21). Calcium is involved in cardiomyocyte repolarization and hypocalcemia is supposed to result in prolongation of QT interval and T-wave inversion (11). Therefore, it could explain the features of hypocalcemic cardiomyopathy that restoration of LVEF and QT interval could be reached with correction of hypocalcemia, and that single conventional therapy for heart failure (e.g., furosemide, which may even aggravate hypocalcemia) is not effective (13, 14). Although normalization of serum calcium concentration was quick, it took 6 months for the complete recovery of myocardial function of our patient. It is reported to take a much longer time for the recovery in some cases (22), indicating that restoring intracellular calcium level is more important.

Hypocalcemia cardiomyopathy in HypoPT is quite rare since most patients would seek for early intervention for hypocalcemia related syndromes. The median interval for development of the disease is reported to be 10 years, ranging from 1.5 months to 36 years (3). Our patient was found to have hypocalcemia, hyperphosphatemia and inappropriate “normal” PTH on admission, and chronic HypoPT was diagnosed. In fact, she developed HypoPT after thyroidectomy 2 years ago, but did not receive regular follow-up. Her symptom of recurrent tetany indicated there were fluctuations of serum calcium which resulted in the development of heart failure. Besides, symptoms of HypoPT do not translate directly to the serum calcium levels and may vary from an asymptomatic state to severe manifestations, which often leads to missed diagnosis and delayed treatment of HypoPT (2). Therefore, it is crucial to monitor and maintain the homeostasis of calcium, phosphate and magnesium for patients with HypoPT, not only to reduce hypocalcemia related syndromes, but also to avoid complications from excessive calcium supplementation. Individuals with unexplained heart failure should be screened for HypoPT, especially for patients with history of neck surgery.

## Conclusion

Hypocalcemic cardiomyopathy is a rare complication of HypoPT. Early correction of hypocalcemia has a dramatic effect in the therapy of heart failure, and cardiac function could recover after normalization of calcium level. Thus, it is indispensable to assess the underlying reversible etiology of each patient with heart failure, and it is pivotal to keep the balance of calcium in HypoPT to reduce the incidence of heart failure development and other complications.

## Data availability statement

The original contributions presented in the study are included in the article/supplementary material, further inquiries can be directed to the corresponding author.

## Ethics statement

Ethical review and approval was not required for the study on human participants in accordance with the local legislation and institutional requirements. The patients/participants provided their written informed consent to participate in this study.

## Author contributions

YW contributed to the work of patients' follow up, data collection, and manuscript draft. XL is responsible for the manuscript revision. All authors contributed to the article and approved the submitted version.

## Funding

This work was supported by Sichuan Science and Technology Program (No. 2022YFQ0009).

## Conflict of interest

The authors declare that the research was conducted in the absence of any commercial or financial relationships that could be construed as a potential conflict of interest.

## Publisher's note

All claims expressed in this article are solely those of the authors and do not necessarily represent those of their affiliated organizations, or those of the publisher, the editors and the reviewers. Any product that may be evaluated in this article, or claim that may be made by its manufacturer, is not guaranteed or endorsed by the publisher.
